# Bladder and upper urinary tract cancers as first and second primary cancers

**DOI:** 10.1002/cnr2.1406

**Published:** 2021-06-11

**Authors:** Guoqiao Zheng, Kristina Sundquist, Jan Sundquist, Asta Försti, Otto Hemminki, Kari Hemminki

**Affiliations:** ^1^ Division of Molecular Genetic Epidemiology German Cancer Research Center (DKFZ) Heidelberg Germany; ^2^ Division of Cancer Epidemiology German Cancer Research Center (DKFZ) Heidelberg Germany; ^3^ Center for Primary Health Care Research Lund University Malmö Sweden; ^4^ Department of Family Medicine and Community Health, Department of Population Health Science and Policy Icahn School of Medicine at Mount Sinai New York New York USA; ^5^ Center for Community‐Based Healthcare Research and Education (CoHRE), Department of Functional Pathology, School of Medicine Shimane University Shimane Japan; ^6^ Hopp Children's Cancer Center (KiTZ) Heidelberg Germany; ^7^ Division of Pediatric Neurooncology, German Cancer Research Center (DKFZ) German Cancer Consortium (DKTK) Heidelberg Germany; ^8^ Department of Surgery University Health Network Toronto Ontario Canada; ^9^ Department of Urology Helsinki University Hospital Helsinki Finland; ^10^ Cancer Gene Therapy Group, Translational Immunology Research Program University of Helsinki Helsinki Finland; ^11^ Biomedical Center, Faculty of Medicine and Biomedical Center in Pilsen Charles University in Prague Pilsen Czech Republic

**Keywords:** cancer etiology, relative risk, renal pelvic cancer, second primary cancer, ureter cancer, urothelial cancer

## Abstract

**Background:**

Previous population‐based studies on second primary cancers (SPCs) in urothelial cancers have focused on known risk factors in bladder cancer patients without data on other urothelial sites of the renal pelvis or ureter.

**Aims:**

To estimate sex‐specific risks for any SPCs after urothelial cancers, and in reverse order, for urothelial cancers as SPCs after any cancer. Such two‐way analysis may help interpret the results.

**Methods:**

We employed standardized incidence ratios (SIRs) to estimate bidirectional relative risks of subsequent cancer associated with urothelial cancers. Patient data were obtained from the Swedish Cancer Registry from years 1990 through 2015.

**Results:**

We identified 46 234 urinary bladder cancers (75% male), 940 ureteral cancers (60% male), and 2410 renal pelvic cancers (57% male). After male bladder cancer, SIRs significantly increased for 9 SPCs, most for ureteral (SIR 41.9) and renal pelvic (17.2) cancers. In the reversed order (bladder cancer as SPC), 10 individual FPCs were associated with an increased risk; highest associations were noted after renal pelvic (21.0) and ureteral (20.9) cancers. After female bladder cancer, SIRs of four SPCs were significantly increased, most for ureteral (87.8) and pelvic (35.7) cancers. Female bladder, ureteral, and pelvic cancers associated are with endometrial cancer.

**Conclusions:**

The risks of recurrent urothelial cancers were very high, and, at most sites, female risks were twice over the male risks. Risks persisted often to follow‐up periods of >5 years, motivating an extended patient follow‐up. Lynch syndrome‐related cancers were associated with particularly female urothelial cancers, calling for clinical vigilance.

## 1 INTRODUCTION

Urothelial carcinomas include bladder cancer (90‐95% of all) and cancer of the upper urinary tract (UUT), of which two‐thirds are located in the renal pelvis and the remaining in the ureter.^1^ Bladder cancer is characterized by male excess, ranging from three‐ to sixfold.^2^, ^3^ International incidence trends have found correlation with the regional smoking prevalence.^2^, ^4^ Bladder cancer incidence in Swedish men has been low and relatively stable at about 20/100 000, while for women, the rate has increased, reaching an incidence of about 6/100 000.^5^, ^6^ Smoking prevalence is not the only explanation to the sex difference in bladder cancer incidence in Sweden, because smoking prevalence in men and women has been approximately equal since 1980 and dropping from 30% to below 10%; around 1950, half of men smoked compared to 10% of women^6^ (www.pnlee.co.uk/downloads/iss/iss-sweden_111024.pdf). UUT cancers show also male excess.^1^ All urothelial cancers share a number of risk factors, including smoking, occupational exposures, family history, and association with Lynch syndrome.^1^, ^7^, ^8^, ^9^ However, while smoking appears to be the main risk factor for bladder cancer compared to Lynch syndrome, the opposite may be the case for UUT cancers.^10^, ^11^ Colorectal cancer and endometrial cancers are traditional hallmarks of Lynch syndrome (www.lscarisk.org).^12^, ^13^ Few guidelines to date suggest urological follow‐up with Lynch patients.^1^

In Sweden, survival has slightly improved for bladder cancer since about 1980.^14^, ^15^, ^16^ In 2012‐2016, the relative 5‐year survival for female bladder cancer was 72% but it was higher, 77% for men (http://www-dep.iarc.fr/NORDCAN/english/frame.asp). Early detection, novel imaging technologies, and improvements in treatment have contributed to positive trends in bladder cancer survival.^5^ At bladder cancer diagnosis, some 20‐25% of patients present with muscle invasive tumors, and the remaining patients have superficial tumors, which can later progress to invasive cancer.^17^ Surgery is the main treatment mode for urothelial cancers. For bladder cancer, non‐muscle‐invasive tumors are transuretherally resected while muscle‐invasive tumors are typically treated with cystectomy; both of which can be supplemented with chemotherapy or immunotherapy.^5^, ^17^ Radiotherapy may be used for bladder preservation.^18^ For UUT cancers, treatment may involve removal of the ipsilateral ureter and kidney.^1^

Improved survival implies that the likelihood of second primary cancers (SPCs) increases. SPCs after bladder cancer show typically high risks of tobacco‐related cancers of the lung and head and neck.^19^, ^20^, ^21^, ^22^ Studies on SPCs in UUT cancer patients are limited, and one of the problems is to distinguish independent SPCs from recurrences.^21^ Recurrence of UUT urothelial cancer into the bladder is relatively common, while seeding from the bladder into UUT is rarer.^1^, ^23^, ^24^, ^25^ Plausible etiologies for SPCs are many, but probably the most important ones are intensive medical surveillance after the diagnosis of first primary cancer (FPC), therapy for FPC, shared genetic or nongenetic risk factors between FPC and SPC and immune dysfunction, or interactions between these.^26^, ^27^, ^28^ As data on the possible risk factors for SPC are usually limited, we have devised a bidirectional analysis as a tool to help etiological search.^26^, ^27^, ^28^, ^29^

In this analysis, we want to define risks for specific subsequent cancers related to bladder and UUT cancers using Swedish nation‐wide data. Risks for SPCs are assessed pair‐wise as FPC and SPC, that is, the standardized incidence ratio (SIR) of lung cancer was assessed after bladder cancer, and, alternatively, SIR of bladder cancer was assessed after lung cancer. The bidirectional analysis will help to distinguish, at least to some extent, the influence of treatment and medical surveillance on SIR because two different cancers are usually treated and diagnosed in different ways. As the previous literature has focused on SPCs after bladder cancer, we hypothesized that the novel type of bidirectional analysis will be able to produce novel qualified data on risks for bladder and UUT cancers.

## 2 MATERIALS AND METHODS

### 2.1 Diagnostic codes and nomenclature

We considered bladder and UUT cancers diagnosed from 1990 to 2015 in the Swedish Cancer Registry using International Classification of Diseases (ICD) version 7 and later codes. The project database is located at the Center for Primary Health Care Research in Malmö, Sweden.

Code 1810 was used for bladder cancers and, of these, 98% are transitional cell carcinomas.^30^ The code for ureter cancer was 1811.^31^ Urethral cancer was not considered because of its rarity and late introduction of a specific diagnostic code. Kidney cancer (ie, renal + pelvis/calyx) was identified with code 180, renal cell cancer (RCC) with code 1800, and pelvic/calyx cancer with 1801. The correctness of classification of UUT cancer in the Cancer Registry has been evaluated, and 93% were found to be correct; the misclassification frequently involved other urinary tract tumors.^32^ In this article, we consider a possible “recurrence” when a second urothelial cancer occurred at the same anatomic site or at another urothelial site, following the practice of the European Association of Urology.^1^

### 2.2 Patient follow‐up periods and methods

Bladder and UUT cancer patients were followed from year 1990 through year 2015 for diagnosis of any common SPC, and, conversely in a reverse order, any common cancer was FPC and bladder, and UUT cancers were SPCs. The other cancers include any of 21 common male and 22 female primary cancers. For a proper bidirectional analysis, we excluded cancers for which 1‐year survival was less than 50% (esophagus, pancreas). Patients were followed for SPCs from the diagnosis of FPC until the end of 2015 or immigration or death, whichever came earliest. Only discordant (different) FPC‐SPC pairs were included without applying any lag time between the two diagnoses. The upper aerodigestive tract (UAT) included the lip, oral cavity, pharynx, and larynx. For skin cancer, only squamous cell carcinoma (SCC) was included. For the risk of “all” cancers, bladder and UUT cancers were excluded. The SIR for “all” cancers was weighed according to person‐years at risk. No latency time was apply between diagnoses of FPC and SPC because in the Swedish Cancer Registry, practically all cancers are histologically verified and thus true cancers.^31^ The application of a latency time would have caused bias because a large number of true SPCs would have been missed.

### 2.3 Calculation of relative risk

Sex‐specific SIRs were calculated to measure the risk of SPCs as the ratio of observed to expected number of cases. For risk of a certain SPC, the expected number of cases was calculated by strata‐specific person years in patients with diagnosis of first primary bladder or UUT cancers, multiplied by strata‐specific incidence rates of the same SPC as FPC in the general population. The strata were specified by sex, 5‐year age group, 5‐year‐calendar period, socioeconomic status, and place of residence. In the reverse analysis, SIRs for second bladder and UUT cancers were calculated in the same way. The two‐tailed 95% confidence intervals (95% CIs) of SIRs were calculated by assuming a Poisson distribution. The expected numbers can be obtained by dividing observed numbers with SIR. The method of SIR calculation is based on indirect standardization, and it is particularly suitable for datasets with small case numbers because the expected numbers are calculated from the large background population, all Sweden in this case.^33^

Bidirectional SIRs for bladder cancer were summarized in forest plots. Pearson correlation coefficient was used to estimate the relation between the bidirectional SIRs for bladder cancer (FPC vs SPC) as well as the SIRs of the common cancers in men and women (men vs women). In addition, we carried out period‐specific analysis by calculating SIRs during 1, 2‐5, and >5 years after first primary cancer diagnosis. All the statistical analyses were performed in SAS 9.4, and forest plot was generated in R 3.3.6. In order to simplify the tables, we did not show data for cancers with less than five cases in any comparison, unless the SIR was significant. The difference between two SIRs was considered significant when their 95% CIs did not overlap. Only significant SIRs were commented on. In the tables, some lines for the urothelial cancers were repeated in the reverse analyses, but they were kept to help comparisons.

## 3 RESULTS

During the follow‐up (inclusion) period of 1990 to 2015, we identified 46 234 bladder cancers, 940 ureter cancers, and 2410 renal pelvic cancers (Table 1). The total number of other cancers considered as SPCs or FPCs was 513 693 for men and 496 600 for women in the concurrent Swedish population of 6.2 million men and 6.2 million women.

**TABLE 1:** Number and median age at diagnosis of the bladder and upper urinary tract cancers identified from 1990 to 2015

Cancer	N (percentage)		Median (lower and upper quartiles) age at diagnosis	
	Male	Female	Male	Female
Bladder	34 676 (75%)	11 558 (25%)	73 (65‐80)	74 (65‐82)
Ureter	564 (60%)	376 (40%)	72 (65‐79)	74 (67‐80)
Renal pelvic	1374 (57%)	1036 (43%)	77 (63‐77)	73 (65‐80)

**FIGURE 1:**
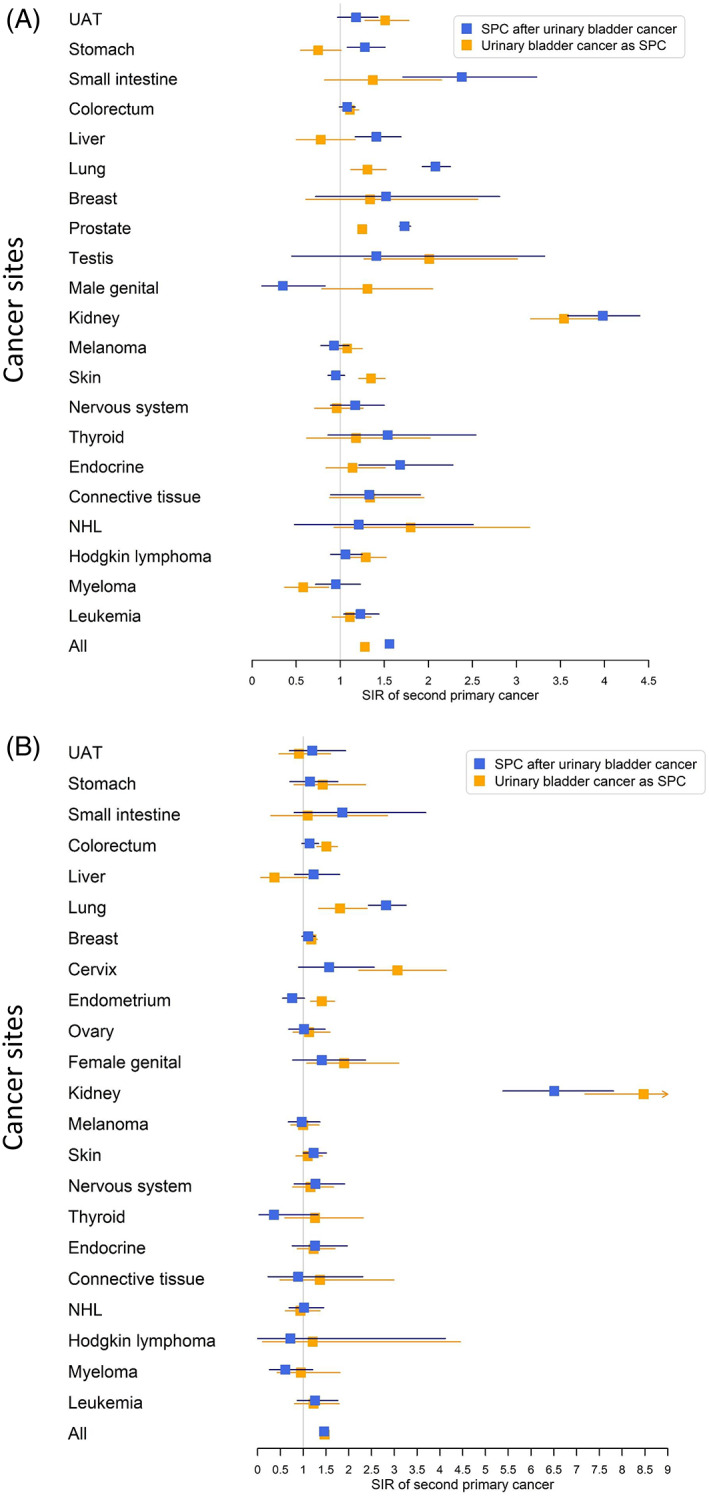
Forest plot on standardized incidence ratios (SIRs) of second primary cancers (SPCs) after urinary bladder cancer and that of urinary bladder cancer after other cancers in men (A) and women (B). SIR for all cancers excluded bladder cancer. SPC, second primary cancer; SIR, standardized incidence ratio; UAT, upper aerodigestive tract; NHL, non‐Hodgkin lymphoma

Bidirectional SIRs for male SPCs for bladder and UUT cancers are shown in Table 2. After bladder cancer, SIRs were significantly increased for 10 SPCs (counting RCC and renal pelvis but not kidney), most for ureteral (SIR 41.9), renal pelvic (17.20), and small intestinal (2.38) cancers. SIR for RCC was 2.20. The overall SIR for any SPC was 1.56. In the reversed order (bladder cancer as SPC), 10 individual FPCs were associated with an increased risk; highest associations were noted after renal pelvic (21.0), ureteral (20.9), and testicular (2.01) cancers. Second bladder cancer risk was 1.45 after RCC. The SIR for all cancers was 1.28. Only five cancers (ureteral, pelvic, RCC, lung and prostate cancers) were bidirectionally associated. For six cancer pairs, the bidirectional SIRs differed significantly (ie, the 95% CIs did not overlap); for ureteral, stomach, lung, RCC, and prostate cancers, the SIRs were higher when these cancers were SPCs than in the reverse order; for skin SCC, the opposite was the case. The data are summarized in Figure 1A.

**TABLE 2:** Male risks of SPCs after bladder, ureteral, and renal pelvic cancers and these cancers as SPCs

Cancer A	Cancer B	Cancer A followed by cancer B				Cancer B followed by cancer A			
		*N*	SIR	95% CI		*N*	SIR	95% CI	
Bladder	UAT	110	1.18	0.97	1.43	143	**1.51**	1.28	1.78
	Stomach	141	**1.28**	1.08	1.51	46	0.75	0.55	1.01
	Small intestine	41	**2.38**	1.71	3.23	19	1.37	0.82	2.15
	CRC	564	1.08	0.99	1.17	521	**1.11**	1.02	1.21
	Liver	117	**1.41**	1.17	1.69	23	0.78	0.50	1.17
	Lung	683	**2.08**	1.93	2.25	174	**1.31**	1.12	1.52
	Breast	10	1.52	0.72	2.81	9	1.34	0.61	2.56
	Prostate	2832	**1.73**	1.67	1.8	2518	**1.25**	1.20	1.30
	Testis	5	1.41	0.45	3.32	23	**2.01**	1.27	3.01
	Male genital	5	0.35	0.11	0.83	19	1.31	0.79	2.05
	Kidney	369	**3.98**	3.58	4.4	309	**3.54**	3.16	3.96
	RCC	140	**2.20**	1.85	2.60	97	**1.45**	1.17	1.77
	Renal pelvis	195	**17.2**	14.9	19.8	199	**21.0**	18.2	24.1
	Ureter	211	**41.9**	36.4	48.0	83	**20.9**	16.6	25.9
	Melanoma	135	0.93	0.78	1.1	174	1.08	0.92	1.25
	Skin SCC	364	0.95	0.86	1.05	337	**1.35**	1.21	1.51
	Nervous system	61	1.17	0.89	1.5	49	0.96	0.71	1.26
	Thyroid	15	1.54	0.86	2.54	13	1.18	0.62	2.02
	Endocrine	41	**1.68**	1.21	2.28	48	1.14	0.84	1.51
	Connective tissue	29	1.33	0.89	1.91	27	1.34	0.88	1.95
	NHL	7	1.21	0.48	2.51	12	1.80	0.93	3.15
	Hodgkin lymphoma	140	1.06	0.89	1.25	141	**1.29**	1.08	1.52
	Myeloma	56	0.95	0.72	1.23	24	0.58	0.37	0.87
	Leukemia	151	**1.23**	1.04	1.44	106	1.11	0.91	1.35
	All	6510	**1.56**	1.53	1.60	4919	**1.28**	1.25	1.32
Ureter	CRC	5	0.85	0.27	2.00	9	1.22	0.55	2.32
	Lung	8	2.12	0.91	4.20	2	0.90	0.09	3.32
	Prostate	20	1.07	0.65	1.66	32	1.00	0.69	1.42
	Kidney	20	**17.9**	10.93	27.74	20	**14.2**	8.67	22.0
	RCC	5	**6.46**	2.04	15.2	2	1.84	0.17	6.76
	Renal pelvis	14	**108.6**	59.2	182.8	18	**129.0**	76.3	204.3
	Bladder	83	**20.9**	16.6	25.9	211	**41.9**	36.4	48.0
	All	167	**2.99**	2.56	3.48	293	**4.46**	3.96	5.00
Renal pelvis	CRC	12	0.85	0.43	1.48	22	1.32	0.83	2.00
	Lung	13	1.39	0.74	2.39	13	1.12	0.40	2.46
	Prostate	54	1.17	0.88	1.53	86	1.19	0.95	1.46
	RCC	5	2.64	0.83	6.22	10	**3.94**	2.70	12.5
	Bladder	199	**21.0**	18.2	24.1	195	**17.2**	14.9	19.8
	Ureter	18	**129.0**	76.3	204.3	14	**108.6**	59.2	182.8
	All	357	**2.73**	2.40	3.03	380	**2.53**	2.28	2.80

*Note*: Bold values show that the 95% CI does not overlap with 1.00.

Abbreviations: CRC, colorectal cancer; *N*, patient number; NHL, non‐Hodgkin lymphoma; RCC, renal cell carcinoma; SCC, squamous cell carcinoma; SIR, standardized incidence ratio; SPC, second primary cancer; UAT, upper aerodigestive tract; 95% CI, 95% confidence interval.

For ureteral cancer, second RCC (6.46), pelvic (108.6), and bladder (20.9) cancers were the only significantly increased SPCs (Table 2). Bladder cancer accounted for half of all SPCs. Ureteral cancer as SPC was associated with renal pelvic (129.0, the highest risk recoded in this study) and bladder (41.9) cancers. After renal pelvic cancer, bladder (21.0) and ureteral (129.0) cancers were increased. Bladder cancer accounted for more than half of all SPCs. In the reverse order, the same cancers and RCC (3.94) showed associations.

Similar analysis is shown for female cancer in Table 3. After bladder cancer, SIRs of five SPCs were significantly increased, including ureteral (87.8), pelvic (35.7), RCC (2.26), lung (2.82), and skin (1.23) cancers. The overall risk was 1.46. In the reverse order (bladder cancer as SPC), nine individual FPCs were associated with an increased risk; SIR of bladder cancer was highest after ureteral (69.9), renal pelvic (60.5), and cervical (3.07) cancers. Notably, SIRs for bladder cancer were increased after all female cancers (except ovarian cancer), including genital (1.90) and endometrial (1.41) cancers. A bidirectional increase in SIRs was found only for pelvic, ureteral, RCC, and lung cancers. The difference between bidirectional SIRs was significant for lung, endometrial, and pelvic cancers; for the latter two cancers, bladder cancer risk was higher when diagnosed as SPC. The overall SIR was 1.47. The data are summarized in Figure 1B.

**TABLE 3:** Female risks of SPCs after bladder, ureteral, and renal pelvic cancers and these cancer as SPCs

Cancer A	Cancer B	Cancer A followed by cancer B				Cancer B followed by cancer A			
		*N*	SIR	95% CI		*N*	SIR	95% CI	
Bladder	UAT	17	1.20	0.70	1.93	12	0.91	0.47	1.6
	Stomach	21	1.15	0.71	1.76	15	1.43	0.80	2.37
	Small intestine	8	1.86	0.80	3.69	4	1.10	0.29	2.85
	CRC	148	1.14	0.97	1.34	183	**1.51**	1.30	1.75
	Liver	27	1.23	0.81	1.8	3	0.37	0.07	1.09
	Lung	181	**2.82**	2.43	3.26	49	**1.81**	1.34	2.4
	Breast	240	1.11	0.97	1.26	382	**1.18**	1.07	1.31
	Cervix	16	1.57	0.90	2.56	43	**3.07**	2.22	4.14
	Endometrium	43	0.76	0.55	1.03	118	**1.41**	1.16	1.69
	Ovary	28	1.02	0.68	1.48	33	1.13	0.78	1.59
	Female genital	14	1.41	0.77	2.37	16	**1.90**	1.08	3.1
	Kidney	116	**6.51**	5.38	7.81	153	**8.47**	7.18	9.92
	RCC	27	**2.26**	1.49	3.29	30	**2.14**	1.44	3.06
	Renal pelvis	82	**35.7**	28.4	44.3	117	**60.5**	50.1	72.6
	Ureter	83	**87.8**	70.0	108.9	49	**69.9**	51.5	92.1
	Melanoma	33	0.97	0.67	1.37	44	1.00	0.73	1.35
	Skin SCC	95	**1.23**	1.00	1.51	61	1.10	0.84	1.42
	Nervous system	23	1.27	0.80	1.91	28	1.16	0.77	1.67
	Thyroid	2	0.36	0.03	1.33	10	1.26	0.60	2.32
	Endocrine	19	1.26	0.76	1.97	38	1.23	0.87	1.7
	Connective tissue	4	0.89	0.23	2.31	6	1.37	0.49	2.99
	NHL	31	1.02	0.69	1.45	26	0.94	0.61	1.37
	Hodgkin lymphoma	1	0.72	0.00	4.12	2	1.21	0.11	4.45
	Myeloma	8	0.61	0.26	1.21	9	0.95	0.43	1.81
	Leukemia	34	1.26	0.87	1.76	27	1.23	0.81	1.79
	All	1329	**1.46**	1.38	1.54	1359	**1.47**	1.39	1.55
Ureter	CRC	7	2.22	0.88	4.60	9	2.13	0.97	4.06
	Lung	6	**4.10**	1.48	8.99	2	1.98	0.19	7.29
	Breast	12	**2.33**	1.20	4.09	17	1.50	0.87	2.4
	Cervix	0	—	—	—	3	**6.46**	1.22	19.1
	Endometrium	3	2.23	0.42	6.61	15	**4.85**	2.71	8.03
	Female genital	1	4.04	0	23.17	2	6.95	0.66	25.6
	Kidney	8	**18.5**	7.89	36.57	15	**22.5**	12.6	37.2
	RCC	1	3.48	0.00	20.0	1	1.94	0	11.1
	Renal pelvis	6	**197.8**	38.8	236.2	13	**191.5**	101.5	328.3
	Bladder	49	**69.9**	51.5	92.1	83	**87.8**	70.0	108.9
	All	94	**3.88**	3.13	4.75	159	**4.74**	4.03	5.54
Renal pelvis	CRC	20	**2.26**	1.38	3.49	9	0.88	0.4	1.68
	Lung	5	1.14	0.36	2.68	7	**2.72**	1.08	5.64
	Breast	13	0.89	0.47	1.52	30	1.06	0.72	1.52
	Cervix	0	—	—	—	2	1.66	0.16	6.10
	Endometrium	4	1.02	0.27	2.64	14	**1.88**	1.02	3.16
	Female genital	2	0.52	0	2.95	0	—	—	—
	RCC	1	1.17	0	6.72	8	**6.32**	2.70	12.5
	Bladder	117	**60.5**	50.1	72.6	82	**35.7**	28.4	44.3
	Ureter	13	**191.5**	101.5	328.3	6	**197.8**	38.8	236.2
	All	205	**3.08**	2.68	3.53	194	**2.35**	2.03	2.70

*Note*: Bold values show that the 95% CI does not overlap with 1.00.

Abbreviations: CRC, colorectal cancer; *N*, patient number; NHL, non‐Hodgkin lymphoma; SCC squamous cell carcinoma; SIR, standardized incidence ratio; SPC, second primary cancer; UAT, upper aerodigestive tract; 95% CI, 95% confidence interval.

After ureteral cancer, increased risks of SPCs were noted for renal pelvic (197.8), bladder (69.9), lung (4.10), and breast (2.33) cancers (Table 2). In the reverse order, SIRs of ureteral cancer were increased after pelvic (191.5), bladder (87.8), cervical (6.46), and endometrial cancers (4.85). After pelvic cancer, second ureteral, bladder, and colorectal (2.26) cancers were increased. In the reverse order, second pelvic cancer was increased after ureteral, bladder, lung (2.72), endometrial (1.88), and RCC (6.32) cancers. Bladder cancer was by far the most common SPC after urethral and pelvic cancers.

We carried out analysis of risk of SPCs depending on the follow‐up time (1 year, 2‐5 years, and >5 years) for male urothelial carcinomas (Table S1). While the SIR was elevated for many SPCs in the first year of follow‐up, that of second lung, RCC, renal pelvic, and ureteral cancers remained elevated throughout the follow‐up time. Of note, second RCC was significant only in the first follow‐up period. Risk of second liver cancer was increased in periods 1 and 2; UAT and stomach cancers and leukemia only in period 2; and NHL only in period 3. Colorectal cancer showed a borderline increase in periods 2 and 3. In the reverse order, second bladder cancer risk was increased after ureteral, prostate, and skin cancers through all periods; association with male genital cancers was significant in periods 1; associations with UAT, colorectal, and lung cancers were significant in periods 2 and 3; and associations with small intestinal, testicular, connective tissue cancers, NHL, and Hodgkin lymphoma were significant in the last period. For ureter and renal pelvic cancers, only bladder and other UUT cancers were significant.

Similar analysis of risk of SPCs for female urothelial carcinomas is shown in Table S2, and association between UUT sites was high throughout the follow‐up time. Risk of second lung cancers remained elevated throughout the follow‐up time. Risk of skin cancer was elevated in period 2 and nervous system cancer in period 3. Risk of breast cancer was elevated after ureteral cancer in period 3. Risk of colorectal cancer was increased after renal pelvic cancer in periods 1 and 2. In the reverse order, bladder cancer as SPC, colorectal and cervical cancers were associated with increased risk through all periods, and renal cell cancer in periods 1 and 2. Associations with breast and endometrial cancers were found in periods 2 and 3, while those with ovarian and lung cancers included period 3. SIR of second ureter cancer was increased after endometrial cancer (6.26) in the last period. Second renal pelvic cancer was increased after colorectal cancer in periods 1 and 2.

Correlation coefficients for the pairwise SIRs were calculated for bladder cancer based on the main sites from Tables 1 and 2 (Table 4). Male vs female Pearson correlation coefficient was 0.92 for SPC after bladder cancer and 0.88 in the reverse order (both *P* < .0001). For SPC vs FPC, the correlation in men was 0.72 (*P* < .0002) and in women it was 0.92 (*P* < .0001).

**TABLE 4:** Correlation analysis between first and second primary cancers in men and women

Type of correlation		No of pairs	Pearson correlation coefficient	*P* value
Men vs Women	Risk of SPC after bladder cancer	18	**0.92**	<.0001
	Risk of bladder cancer as SPC	18	**0.88**	<.0001
Risk of SPC vs risk as FPC	Bladder in men	21	**0.72**	.0002
	Bladder in women	20	**0.92**	<.0001

*Note*: Bold values show that the 95% CI does not overlap with 1.00.

Abbreviations: FPC, first primary cancer; SPC, second primary cancer.

## 4 DISCUSSION

### 4.1 Novel approach

The observation with direct clinical implications was the very high mutual associations of bladder, ureteral, and renal pelvic cancers with each other as FPC and SPCs. Although the recurrence risk is well known in urology, it has never been defined before with the present precision.^1^, ^34^, ^35^ Among all cancer considered, relative risks of 10 cancers were significant as SPCs after bladder cancer, and second bladder cancer was significant after 10 FPCs, only five male cancers showed a bidirectional association. For women, four cancers were bidirectionally increased. Bidirectional associations of bladder cancer were shared for men and women for ureteral, renal pelvic, RCC, and lung cancers. The correlation analysis showed high concordance (*P* < .00021) between men and women when SPCs were compared after bladder cancer or in the reverse order, bladder cancer as SPC. Although previous studies have identified risks of smoking‐related SPCs after bladder cancer, the novel results in this study define bidirectional risks between many sites not related to smoking.^19^, ^20^, ^21^, ^22^

In the below discussion, we compare the consistency of the results internally and externally (with published literature) and speculate about the putative mechanisms, keeping in mind the limitations of observational epidemiology.

### 4.2 Urological sites

Urothelial carcinomas have been described as a pan‐urothelial disease with a propensity to recur throughout these sites.^24^, ^36^ As with many other cancers, even intratumoral heterogenicity has been described.^37^ However, a recent literature review concluded that most recurrent urothelial tumors are monoclonal which would imply that the mechanism of spread would be intraluminal seeding or intraepithelial migration.^24^ These in turn would imply that the direction of urine flow and anatomic vicinity would play a role. In a Spanish study, concomitant primary urothelial tumors were found in 17% of the patients.^36^ The likelihood of finding a concomitant bladder cancer increased by anatomic location of the primary tumor, being 10, 18, and 33% in patients with primary caliceal/renal pelvic, upper ureteral, and lower ureteral cancers, in line with the above predictions. The pan‐urothelial disease is the likely explanation why the risks among urothelial sites far exceeded those between urothelial and nonurothelial sites.

In the present study, the male risk of second bladder cancer was equally high (SIR 21) when renal pelvic or ureteral cancers were FPCs, but, for women, the risks were slightly higher after ureteral (69.9) than after pelvic (60.5) cancers. Most urothelial cancer associations were higher in women than in men. The female SIRs were more than doubled and significantly higher compared to male rates for FCP‐SPC pairs of bladder‐pelvis (SIR in women 35.7), pelvis‐bladder (60.5), bladder‐ureter (87.8), and ureter‐bladder (69.9). Although the risks were highest during the year of diagnosis, they persisted often to follow‐up period of >5 years (conditional on patient survival). These risks were exquisitely high and clinically relevant, motivating an extended patient follow‐up. Bladder cancer was by far the most common SPC after first ureteral and renal pelvic cancers, accounting for at least half of all SPCs.

Of urological interest is also the possible SPC risks between bladder, RCC, and prostate cancers. The SIRs for second male RCC and prostate cancer were 2.20 and 1.73, respectively, and prostate cancer accounted for 44% of all SPCs after bladder cancer. Male SIRs for bladder cancer as SPC after these cancers were more modest, 1.45 and 1.25, respectively, but second bladder cancer following prostate cancer accounted for 51% of all bladder cancers as SPC. SIR for second RCC in women was 2.26 and in the reverse order 2.14. However, when analyzed by follow‐up time, the risk for second prostate cancer was increased only in the year of bladder cancer diagnosis, which suggests that the excess risk was due to surveillance bias. The conclusion about second RCC was similar for men but for women remained unclear. Thus, the study provided no evidence for association of urothelial cancers and RCC or prostate cancer.

### 4.3 Urological with other sites

For the other cancers, we considered the multiple independent results for consistency in order to conclude about true associations. These included results between both sexes in bidirectional analysis and, in order to exclude surveillance bias, only considering the follow‐up times 2‐5 years and >5 years after diagnosis of FPC (thus yielding eight comparisons for cancers diagnosed in both sexes). Lung cancer was significant in seven associations, colorectal cancer in four, UAT, prostate, and skin cancers in three, and breast, cervical, and endometrial cancers and non‐Hodgkin lymphoma in two. The associations with lung and upper aerodigestive tract cancers were most likely related to smoking and that of prostate cancer to residual surveillance bias. The risk for cervical cancer (and, for female, genital cancer) was most likely an example of long‐term surveillance bias, considering risk reduction along the follow‐up time, anatomic vicinity, and known viral etiology. We have previously observed such long‐term surveillance bias for urolithiasis with various cancers, particularly for cancers in anatomic vicinity.^38^ In another study on female vaginal and vulvar cancer, which share the viral etiology with cervical cancer, we have shown that the risk for second bladder cancer was 1.88 (*N* = 45, 95% CI 1.35‐2.44).^39^

The associations of bladder, ureteral, and renal pelvic cancers with colorectal, small intestinal, endometrial, and ovarian cancers may signal the contribution of genetic factors. Bladder cancer is considered a minor component in Lynch syndrome (hereditary nonpolyposis colorectal cancer syndrome), caused by germline mutations in mismatch repair genes.^7^ It is plausible that the increased risks of second ureteral (4.85) and pelvic (1.88) cancers after endometrial cancer were contributed by Lynch syndrome.^10^, ^11^ The reason for the higher associations of the Lynch syndrome‐related cancers in women compared to men may be due to the lower female population incidence of urothelial cancers, particularly of bladder cancer. The difference in population incidence was probably in part related to historic higher smoking level in men compared to women, as pointed out in Introduction. However, as in all population level studies, we lacked the definitive genetic evidence, but want to remind that the diagnostic guidelines based on the Amsterdam or Bethesda criteria emphasize family history.^40^

Among the remaining associations with bladder cancer, using the above criteria for consistency, skin SCC had three and non‐Hodgkin lymphoma two associations. The common denominator for these two cancer is that they are a hallmark of immune disturbance and are vastly elevated in immune suppressed patients.^41^, ^42^ It is thus plausible that immune dysfunctions may contribute to the associations of bladder cancers with skin cancer and NHL.^41^ Finally, there were solitary associations for male bladder cancers as SPC, which emerged >5 years after diagnosis of testicular cancer and Hodgkin lymphoma. These are early‐onset cancers treated with intense chemotherapy and/or radiotherapy with high risk of SPCs, including bladder cancer.^43^, ^44^, ^45^

### 4.4 Strengths and limitations

This study has a number of strengths, the foremost being a nation‐wide coverage and access to a high‐level cancer registry data.^31^, ^46^ Because of linkage to the censuses, we had information on socio‐economic and residential background data covering the whole population, which is unique in nation‐wide studies. Socio‐economic data are highly correlated with lung cancer incidence in Sweden and thus provide a proxy of smoking level.^47^, ^48^, ^49^ This kind of studies with numerous comparisons will produce some significant associations by chance. Assessing both sexes separately and comparing the consistency was helpful in avoiding chance findings. The bidirectional design is another strength in helping to interpret the associations. Ureteral and renal pelvic cancers are rare, and all related case numbers were low, affording low statistical power. The major limitation was that data on treatment were lacking, but we assume that surgery has been the primary treatment for bladder and UUT cancers.

## 5 CONCLUSIONS

We showed that many cancers were associated with bladder cancer as SPCs or, in the reverse order, bladder cancer as SPC. The risks of recurrence of urothelial cancers were very high, and, at most sites, female risks were twice over the male risks. Risks persisted often through follow‐up periods of >5 years, motivating an extended patient follow‐up. Apart from smoking‐related cancers, immune dysfunction was suggested to contribute to associations with skin cancer and non‐Hodgkin lymphoma. Lynch syndrome‐related cancers were associated with urothelial cancers, providing population‐level justification for diagnostic and management recommendations.^1^

## AUTHOR CONTRIBUTIONS

**Kristina Sundquist:** Funding acquisition; investigation; project administration; resources; software; visualization; writing‐review & editing; Funding and administration. **Jan Sundquist:** Funding acquisition; investigation; project administration; resources; software; visualization; writing‐review & editing; Acquisition of data; Funding and administration. **Asta Försti:** Formal analysis; investigation; validation; visualization; writing‐review & editing; Statistical analysis and interpretation. **Otto Hemminki:** Formal analysis; investigation; validation; visualization; writing‐review & editing; Statistical analysis and interpretation. **Guoqiao Zheng:** Design; Statistical analysis and interpretation. **Kari Hemminki:** Design; Funding and administration. **All authors:** Manuscript writing; Approval of the final text.

## CONFLICT OF INTEREST

None.

## ETHICAL STATEMENT

The study was approved on 25 January 2013, by the Regional Ethical Review Board in Lund without requirement for informed consent (registration number 2012/795). Instead, the Ethical Review Board obliged us to advertise in the newspapers in order to inform the public that their data would be used for secondary purposes. After that, around 40 individuals were excluded (≈40 excluded individuals/≈10 million individuals in the Swedish population).

## Supporting information


**TABLE S1** Male risks of SPCs after bladder, ureteral or renal pelvic cancers and these cancer as SPCs stratified by follow‐up time after first primary cancer diagnosis
**TABLE S2** Female risks of SPCs after bladder, ureteral or renal pelvic cancers and these cancer as SPCs stratified by follow‐up time after first primary cancer diagnosis

## Data Availability

DATA AVAILABILITY The data that support the findings of this study are available from Lund University but restrictions apply to the availability of these data, which were used under license for the current study and so are not publicly available.
