# Supplementary material for: Bladder and upper urinary tract cancers as first and second primary cancers
Source: Cancer Rep (Hoboken). 2021 Jun 11;4(6):e1406. doi: 10.1002/cnr2.1406 (PMC8714543; doi:10.1002/cnr2.1406)
Supplement: Supplementary file 1 — TABLE S1 Male risks of SPCs after bladder, ureteral or renal pelvic cancers and these cancer as SPCs stratified by follow‐up time after first primary cancer diagnosis TABLE S2 Female risks of SPCs after bladder, ureteral or renal pelvic cancers and these cancer as SPCs stratified by follow‐up time after first primary cancer diagnosis [file CNR2-4-e1406-s001.docx]

Supplementary Table 1. Male risks of SPCs after bladder, ureteral or renal pelvic cancers and these cancer as SPCs stratified by follow-up time after first primary cancer diagnosis

| **Cancer A** | **Cancer B** | 1 year | | | | 2-5 years | | | | > 5 years | | | |
| --- | --- | --- | --- | --- | --- | --- | --- | --- | --- | --- | --- | --- | --- |
|  |  | N | SIR | 95% CI | | N | SIR | 95% CI | | N | SIR | 95% CI | |
| **Cancer A followed by cancer B** | | | | | | | | | | | | | |
| **Bladder** | UAT | 21 | 0.78 | 0.48 | 1.20 | 49 | **1.56** | 1.15 | 2.06 | 40 | 1.15 | 0.82 | 1.57 |
|  | Stomach | 37 | 1.11 | 0.78 | 1.53 | 56 | **1.49** | 1.12 | 1.93 | 48 | 1.24 | 0.91 | 1.64 |
|  | Small intestine | 25 | **5.24** | 3.39 | 7.75 | 7 | 1.23 | 0.49 | 2.54 | 9 | 1.33 | 0.60 | 2.54 |
|  | CRC | 134 | 0.94 | 0.78 | 1.11 | 196 | 1.13 | 0.98 | 1.31 | 234 | 1.13 | 0.99 | 1.29 |
|  | Liver | 40 | **1.61** | 1.15 | 2.20 | 42 | **1.50** | 1.08 | 2.03 | 35 | 1.17 | 0.81 | 1.63 |
|  | Lung | 163 | **1.71** | 1.46 | 2.00 | 244 | **2.22** | 1.95 | 2.51 | 276 | **2.25** | 1.99 | 2.53 |
|  | Breast | 3 | 1.69 | 0.32 | 5.01 | 2 | 0.92 | 0.09 | 3.38 | 5 | 1.91 | 0.60 | 4.49 |
|  | Prostate | 1703 | **3.76** | 3.58 | 3.94 | 538 | 1.00 | 0.92 | 1.09 | 591 | **0.92** | 0.85 | 0.99 |
|  | Testis | 3 | 2.76 | 0.52 | 8.16 | 0 | - | - | - | 2 | 1.66 | 0.16 | 6.10 |
|  | Male genital | 3 | 0.79 | 0.15 | 2.35 | 0 | - | - | - | 2 | 0.35 | 0.03 | 1.29 |
|  | Kidney | 175 | **6.43** | 5.52 | 7.46 | 89 | **2.83** | 2.27 | 3.48 | 105 | **3.07** | 2.51 | 3.72 |
|  | RCC | 83 | **4.33** | 3.45 | 5.38 | 30 | 1.37 | 0.93 | 1.96 | 27 | 1.20 | 0.79 | 1.74 |
|  | Renal pelvis | 72 | **22.8** | 17.9 | 28.8 | 53 | **14.2** | 10.6 | 18.5 | 70 | **15.8** | 12.3 | 19.9 |
|  | Ureter | 82 | **57.4** | 45.6 | 76.2 | 71 | **42.0** | 32.8 | 52.9 | 58 | **30.3** | 23.0 | 39.2 |
|  | Melanoma | 28 | 0.75 | 0.5 | 1.08 | 49 | 1.04 | 0.77 | 1.38 | 58 | 0.95 | 0.72 | 1.22 |
|  | Skin | 66 | **0.72** | 0.55 | 0.91 | 115 | 0.96 | 0.79 | 1.15 | 183 | 1.07 | 0.92 | 1.24 |
|  | Nervous system | 14 | 0.90 | 0.49 | 1.51 | 21 | 1.18 | 0.73 | 1.80 | 26 | 1.37 | 0.90 | 2.02 |
|  | Thyroid | 4 | 1.42 | 0.37 | 3.66 | 5 | 1.52 | 0.48 | 3.57 | 6 | 1.65 | 0.60 | 3.62 |
|  | Endocrine | 19 | **2.63** | 1.58 | 4.11 | 8 | 0.95 | 0.41 | 1.89 | 14 | 1.59 | 0.87 | 2.68 |
|  | Connective tissue | 9 | 1.49 | 0.67 | 2.83 | 11 | 1.52 | 0.75 | 2.73 | 9 | 1.06 | 0.48 | 2.02 |
|  | NHL | 26 | 0.71 | 0.47 | 1.05 | 46 | 1.06 | 0.77 | 1.41 | 68 | **1.30** | 1.01 | 1.64 |
|  | Hodgkin lymphoma | 1 | 0.58 | 0 | 3.32 | 3 | 1.52 | 0.29 | 4.49 | 3 | 1.45 | 0.27 | 4.30 |
|  | Myeloma | 14 | 0.85 | 0.46 | 1.43 | 14 | 0.71 | 0.39 | 1.20 | 28 | 0.81 | 0.11 | 1.77 |
|  | Leukemia | 44 | 1.32 | 0.96 | 1.77 | 54 | **1.34** | 1.01 | 1.75 | 53 | 1.08 | 0.81 | 1.41 |
|  | ALL | 2735 | **2.37** | 2.28 | 2.46 | 1762 | **1.28** | 1.22 | 1.34 | 2013 | **1.23** | 1.18 | 1.29 |
| **Ureter** | Lung | 0 | - | - | - | 4 | 3.39 | 0.88 | 8.77 | 4 | 3.16 | 0.82 | 8.18 |
|  | Prostate | 6 | 1.01 | 0.36 | 2.21 | 7 | 1.20 | 0.48 | 2.48 | 7 | 1.02 | 0.40 | 2.11 |
|  | Kidney | 16 | **40.3** | 23.0 | 65.7 | 2 | 5.65 | 0.53 | 20.8 | 2 | 5.48 | 0.52 | 20.2 |
|  | RCC | 5 | **18.2** | 5.74 | 42.8 | 0 | - | - | - | 0 | - | - | - |
|  | Renal pelvis | 10 | **230.0** | 109.3 | 423.8 | 2 | **49.8** | 4.70 | 183.3 | 2 | **44.3** | 4.17 | 162.8 |
|  | Bladder | 59 | **42.3** | 32.2 | 54.6 | 19 | **16.0** | 9.64 | 25.1 | 5 | **3.58** | 1.13 | 8.42 |
|  | ALL | 90 | **4.70** | 3.78 | 5.77 | 41 | **2.43** | 1.74 | 3.30 | 36 | **1.82** | 1.28 | 2.53 |
| **Renal pelvis** | CRC | 1 | 0.22 | 0 | 1.29 | 6 | 1.39 | 0.50 | 3.04 | 6 | 0.96 | 0.35 | 2.11 |
|  | Lung | 3 | 0.93 | 0.18 | 2.76 | 4 | 1.43 | 0.37 | 3.7 | 8 | 2.09 | 0.89 | 4.15 |
|  | Prostate | 14 | 1.02 | 0.56 | 1.72 | 11 | 0.79 | 0.39 | 1.43 | 31 | 1.46 | 0.99 | 2.07 |
|  | RCC | 4 | **6.12** | 1.59 | 15.83 | 0 | - | - | - | 1 | 1.54 | 0 | 8.86 |
|  | Bladder | 129 | **40.4** | 33.71 | 47.99 | 62 | **21.83** | 16.74 | 28 | 8 | 2.32 | 0.99 | 4.59 |
|  | Ureter | 15 | **56.2** | 31.3 | 92.8 | 3 | 69.0 | 13.0 | 204.2 | 0 | - | - | - |
|  | All | 183 | **4.22** | 3.64 | 4.88 | 110 | **2.79** | 2.29 | 3.36 | 64 | **1.34** | 1.03 | 1.71 |
| **Cancer B followed by cancer A** | | | | | | | | | | | | | |
| **Bladder** | UAT | 27 | 0.99 | 0.65 | 1.44 | 54 | **1.79** | 1.34 | 2.34 | 62 | **1.67** | 1.28 | 2.14 |
|  | Stomach | 17 | **0.53** | 0.31 | 0.85 | 13 | 0.89 | 0.47 | 1.52 | 16 | 1.11 | 0.64 | 1.81 |
|  | Small intestine | 3 | 0.66 | 0.12 | 1.96 | 6 | 1.32 | 0.47 | 2.88 | 10 | **2.11** | 1.01 | 3.90 |
|  | CRC | 135 | 0.92 | 0.77 | 1.09 | 190 | **1.19** | 1.03 | 1.38 | 196 | **1.20** | 1.04 | 1.38 |
|  | Liver | 11 | **0.51** | 0.25 | 0.92 | 8 | 1.55 | 0.66 | 3.07 | 4 | 1.45 | 0.38 | 3.74 |
|  | Lung | 93 | 1.03 | 0.83 | 1.27 | 41 | **1.54** | 1.10 | 2.09 | 40 | **2.44** | 1.74 | 3.33 |
|  | Breast | 5 | 2.74 | 0.87 | 6.45 | 2 | 0.82 | 0.08 | 3.02 | 2 | 0.82 | 0.08 | 3.03 |
|  | Prostate | 843 | **1.62** | 1.51 | 1.73 | 851 | **1.12** | 1.05 | 1.20 | 824 | **1.11** | 1.04 | 1.19 |
|  | Testis | 3 | 2.55 | 0.48 | 7.54 | 1 | 0.47 | 0 | 2.68 | 19 | **2.33** | 1.40 | 3.65 |
|  | Male genital | 9 | **2.27** | 1.03 | 4.32 | 6 | 1.26 | 0.45 | 2.76 | 4 | 0.69 | 0.18 | 1.79 |
|  | Kidney | 180 | **6.65** | 5.72 | 7.70 | 85 | **3.09** | 2.47 | 3.83 | 44 | 1.35 | 0.98 | 1.81 |
|  | RCC | 42 | **2.17** | 1.56 | 2.94 | 20 | **0.96** | 0.58 | 1.48 | 35 | 1.31 | 0.91 | 1.82 |
|  | Renal pelvis | 129 | **40.4** | 33.7 | 48.0 | 62 | **21.8** | 16.7 | 28.0 | 8 | 2.32 | 0.99 | 4.59 |
|  | Ureter | 59 | **42.3** | 32.2 | 54.6 | 19 | **16.0** | 9.64 | 25.1 | 5 | **3.58** | 1.13 | 8.42 |
|  | Melanoma | 42 | 1.09 | 0.79 | 1.48 | 51 | 0.99 | 0.74 | 1.3 | 81 | 1.13 | 0.90 | 1.40 |
|  | Skin | 98 | **1.24** | 1.00 | 1.51 | 127 | **1.37** | 1.15 | 1.64 | 112 | **1.45** | 1.19 | 1.74 |
|  | Nervous system | 11 | 0.70 | 0.35 | 1.25 | 8 | 0.66 | 0.28 | 1.31 | 30 | 1.28 | 0.86 | 1.83 |
|  | Thyroid | 2 | 0.74 | 0.07 | 2.72 | 5 | 1.58 | 0.50 | 3.71 | 6 | 1.16 | 0.42 | 2.54 |
|  | Endocrine | 10 | 1.32 | 0.63 | 2.44 | 15 | 1.27 | 0.71 | 2.1 | 23 | 1.01 | 0.64 | 1.52 |
|  | Connective tissue | 5 | 0.82 | 0.26 | 1.93 | 6 | 0.95 | 0.34 | 2.08 | 16 | **2.05** | 1.17 | 3.34 |
|  | NHL | 37 | 1.01 | 0.71 | 1.39 | 47 | 1.29 | 0.95 | 1.72 | 57 | **1.56** | 1.18 | 2.03 |
|  | Hodgkin lymphoma | 1 | 0.61 | 0 | 3.49 | 3 | 1.60 | 0.30 | 4.73 | 8 | **2.54** | 1.08 | 5.03 |
|  | Myeloma | 9 | **0.50** | 0.23 | 0.95 | 11 | 0.67 | 0.33 | 1.21 | 4 | 0.58 | 0.15 | 1.49 |
|  | Leukemia | 32 | 0.97 | 0.66 | 1.37 | 36 | 1.11 | 0.78 | 1.54 | 38 | 1.28 | 0.91 | 1.76 |
|  | ALL | 1681 | **1.42** | 1.35 | 1.49 | 1618 | **1.22** | 1.17 | 1.29 | 1620 | **1.22** | 1.16 | 1.28 |
| **Ureter** | Lung | 1 | 0.65 | 0 | 3.73 | 0 | - | - | - | 1 | 4.01 | 0 | 23.0 |
|  | Prostate | 13 | 1.51 | 0.80 | 2.59 | 9 | 0.74 | 0.34 | 1.41 | 10 | 0.89 | 0.43 | 1.65 |
|  | Kidney | 17 | **37.6** | 21.8 | 60.3 | 3 | **6.68** | 1.26 | 19.78 |  | **1.22** | 1.16 | 1.28 |
|  | RCC | 2 | 6.03 | 0.57 | 22.2 | 0 | - | - | - | 0 | - | - | - |
|  | Renal pelvis | 15 | **56.2** | 31.3 | 92.8 | 3 | 69.0 | 13.0 | 204.2 | 0 | - | - | - |
|  | Bladder | 82 | **57.4** | 45.6 | 76.2 | 71 | **42.0** | 32.8 | 52.9 | 58 | **30.3** | 23.0 | 39.2 |
|  | ALL | 124 | **5.90** | 4.91 | 7.03 | 90 | **3.94** | 3.17 | 4.85 | 79 | **3.61** | 2.86 | 4.50 |
| **Renal pelvis** | CRC | 1 | 0.22 | 0 | 1.29 | 6 | 1.39 | 0.50 | 3.04 | 6 | 0.96 | 0.35 | 2.11 |
|  | Lung | 3 | 0.93 | 0.18 | 2.76 | 4 | 1.43 | 0.37 | 3.7 | 8 | 2.09 | 0.89 | 4.15 |
|  | Prostate | 14 | 1.02 | 0.56 | 1.72 | 11 | 0.79 | 0.39 | 1.43 | 31 | 1.46 | 0.99 | 2.07 |
|  | RCC | 4 | **6.12** | 1.59 | 15.83 | 0 | - | - | - | 1 | 1.54 | 0 | 8.86 |
|  | Bladder | 72 | **22.8** | 17.9 | 28.8 | 53 | **14.2** | 10.6 | 18.5 | 70 | **15.8** | 12.3 | 19.9 |
|  | Ureter | 10 | **230.0** | 109.3 | 423.8 | 2 | **49.8** | 4.70 | 183.3 | 2 | **44.3** | 4.17 | 162.8 |
|  | All | 144 | **3.02** | 2.55 | 3.56 | 103 | **1.99** | 1.62 | 2.42 | 133 | **2.63** | 2.20 | 3.12 |

SPC, second primary cancer, N= patient number, SIR standardized incidence ratio, 95%CI 95% confidence interval, RCC renal cell carcinoma, UAT, upper aerodigestive tract, CRC, colorectal cancer, NHL, non-Hodgkin lymphoma

Supplementary Table 2. Female risks of SPCs after bladder, ureteral or renal pelvic cancers and these cancer as SPCs stratified by follow-up time after first primary cancer diagnosis

| **Cancer A** | **Cancer B** | 1 year | | | | 2-5 years | | | | > 5 years | | | |
| --- | --- | --- | --- | --- | --- | --- | --- | --- | --- | --- | --- | --- | --- |
|  |  | N | SIR | 95% CI | | N | SIR | 95% CI | | N | SIR | 95% CI | |
| **Cancer A followed by cancer B** | | | | | | | | | | | | | |
| Bladder | UAT | 5 | 1.31 | 0.41 | 3.07 | 5 | 1.12 | 0.35 | 2.64 | 7 | 1.19 | 0.47 | 2.47 |
|  | Stomach | 6 | 1.06 | 0.38 | 2.33 | 9 | 1.54 | 0.70 | 2.94 | 6 | 0.88 | 0.32 | 1.93 |
|  | Small intestine | 3 | 2.50 | 0.47 | 7.41 | 0 | - | - | - | 5 | 2.87 | 0.90 | 6.74 |
|  | CRC | 33 | 0.94 | 0.64 | 1.31 | 48 | 1.18 | 0.87 | 1.57 | 67 | 1.25 | 0.97 | 1.59 |
|  | Liver | 7 | 1.01 | 0.40 | 2.10 | 10 | 1.42 | 0.68 | 2.63 | 10 | 1.26 | 0.60 | 2.33 |
|  | Lung | 35 | **2.01** | 1.40 | 2.80 | 63 | **3.12** | 2.40 | 4.00 | 83 | **3.13** | 2.49 | 3.88 |
|  | Breast | 66 | 1.12 | 0.87 | 1.42 | 78 | 1.12 | 0.88 | 1.40 | 96 | 1.10 | 0.89 | 1.34 |
|  | Cervix | 8 | **2.66** | 1.14 | 5.27 | 7 | 2.11 | 0.84 | 4.37 | 1 | 0.26 | 0 | 1.48 |
|  | Endometrium | 7 | **0.46** | 0.18 | 0.95 | 19 | 1.05 | 0.63 | 1.65 | 17 | 0.74 | 0.43 | 1.18 |
|  | Ovary | 11 | 1.36 | 0.68 | 2.44 | 9 | 1.00 | 0.45 | 1.90 | 8 | 0.77 | 0.33 | 1.53 |
|  | Female genital | 4 | 1.47 | 0.38 | 3.79 | 7 | 2.29 | 0.91 | 4.74 | 3 | 0.72 | 0.14 | 2.14 |
|  | Kidney | 60 | **11.4** | 8.68 | 14.6 | 24 | **4.16** | 2.66 | 6.19 | 32 | **4.72** | 3.23 | 6.67 |
|  | RCC | 18 | **4.94** | 2.92 | 7.81 | 6 | 1.52 | 0.55 | 3.33 | 3 | 0.69 | 0.13 | 2.04 |
|  | Renal pelvis | 39 | **61.1** | 43.4 | 83.6 | 18 | **24.8** | 14.7 | 39.3 | 25 | **26.8** | 17.3 | 39.6 |
|  | Ureter | 46 | **181.0** | 132.4 | 241.5 | 16 | **53.8** | 30.6 | 87.5 | 21 | **53.4** | 33.0 | 81.8 |
|  | Melanoma | 11 | 1.27 | 0.63 | 2.28 | 10 | 0.94 | 0.45 | 1.74 | 12 | 0.82 | 0.42 | 1.43 |
|  | Skin | 16 | 0.88 | 0.50 | 1.44 | 36 | **1.60** | 1.12 | 2.21 | 43 | 1.18 | 0.85 | 1.59 |
|  | Nervous system | 4 | 0.76 | 0.20 | 1.96 | 4 | 0.67 | 0.17 | 1.74 | 15 | **2.19** | 1.22 | 3.62 |
|  | Thyroid | 0 | - | - | - | 1 | 0.56 | 0 | 3.23 | 1 | 0.46 | 0 | 2.62 |
|  | Endocrine | 5 | 1.11 | 0.35 | 2.61 | 6 | 1.18 | 0.43 | 2.59 | 8 | 1.44 | 0.62 | 2.86 |
|  | Connective tissue | 2 | 1.58 | 0.15 | 5.80 | 1 | 0.70 | 0 | 4.04 | 1 | 0.56 | 0 | 3.22 |
|  | NHL | 3 | 0.36 | 0.07 | 1.05 | 11 | 1.15 | 0.57 | 2.06 | 17 | 1.38 | 0.80 | 2.21 |
|  | Hodgkin lymphoma | 0 | - | - | - | 0 | - | - | - | 1 | 1.88 | 0 | 10.75 |
|  | Myeloma | 2 | 0.54 | 0.05 | 2.00 | 3 | 0.72 | 0.14 | 2.14 | 3 | 0.57 | 0.11 | 1.68 |
|  | Leukemia | 5 | 0.69 | 0.22 | 1.62 | 13 | 1.55 | 0.82 | 2.65 | 16 | 1.42 | 0.81 | 2.30 |
|  | ALL | 377 | **1.52** | 1.37 | 1.68 | 423 | **1.46** | 1.32 | 1.61 | 529 | **1.42** | 1.30 | 1.55 |
| **Ureter** | Lung | 2 | 3.75 | 0.35 | 13.8 | 2 | 4.00 | 0.38 | 14.7 | 2 | 4.66 | 0.44 | 17.2 |
|  | Breast | 1 | 0.59 | 0 | 3.41 | 4 | 2.31 | 0.60 | 5.97 | 7 | **4.05** | 1.61 | 8.39 |
|  | Endometrium | 0 | - | - | - | 2 | 4.35 | 0.41 | 16.0 | 1 | 2.39 | 0 | 13.7 |
|  | Female genital | 1 | 12.8 | 0.01 | 73.1 | 0 | - | - | - | 0 | - | - | - |
|  | Kidney | 8 | **48.0** | 20.5 | 95.1 | 0 | - | - | - | 0 | - | - | - |
|  | RCC | 1 | 8.90 | 0.00 | 22.2 | 0 | - | - | - | 0 | - | - | - |
|  | Renal pelvis | 6 | **308.4** | 111.0 | 675.8 | 0 | - | - | - | 0 | - | - | - |
|  | Bladder | 35 | **140.1** | 97.5 | 195.0 | 9 | **38.9** | 17.6 | 74.1 | 5 | **22.5** | 7.09 | 52.8 |
|  | ALL | 53 | **6.13** | 4.59 | 8.02 | 24 | **3.00** | 1.92 | 4.47 | 17 | **2.24** | 1.30 | 3.59 |
| **Renal pelvis** | CRC | 8 | **2.90** | 1.24 | 5.74 | 8 | **3.11** | 1.33 | 6.15 | 4 | 1.13 | 0.29 | 2.93 |
|  | Lung | 1 | 0.69 | 0 | 3.95 | 1 | 0.78 | 0 | 4.48 | 3 | 1.81 | 0.34 | 5.36 |
|  | Breast | 4 | 0.88 | 0.23 | 2.28 | 3 | 0.68 | 0.13 | 2 | 6 | 1.06 | 0.38 | 2.33 |
|  | Cervix | 0 | - | - | - | 0 | - | - | - | 0 | - | - | - |
|  | Endometrium | 1 | 0.81 | 0 | 4.65 | 0 | - | - | - | 3 | 1.98 | 0.37 | 5.86 |
|  | Female genital | 1 | 4.87 | 0 | 27.92 | 1 | 5.35 | 0 | 30.64 | 0 | - | - | - |
|  | RCC | 1 | 3.27 | 0 | 18.74 | 0 | - | - | - | 0 | - | - | - |
|  | Bladder | 76 | **120.4** | 94.8 | 150.7 | 32 | **58.4** | 39.9 | 82.5 | 9 | **12.0** | 5.42 | 22.8 |
|  | Ureter | 9 | **428** | 194.1 | 816.6 | 3 | **150.1** | 28.3 | 444.2 | 1 | 37.2 | 0.01 | 213.1 |
|  | All | 110 | **4.99** | 4.10 | 6.02 | 59 | **3.04** | 2.31 | 3.92 | 36 | **1.44** | 1.01 | 1.99 |
| **Cancer B followed by cancer A** | | | | | | | | | | | | | |
| **Bladder** | UAT | 3 | 0.80 | 0.15 | 2.37 | 2 | 0.48 | 0.05 | 1.76 | 7 | 1.34 | 0.53 | 2.77 |
|  | Stomach | 5 | 0.97 | 0.31 | 2.27 | 6 | 2.36 | 0.85 | 5.17 | 4 | 1.45 | 0.38 | 3.75 |
|  | Small intestine | 0 | - | - | - | 1 | 0.86 | 0 | 4.95 | 3 | 2.27 | 0.43 | 6.71 |
|  | CRC | 50 | **1.42** | 1.05 | 1.87 | 63 | **1.61** | 1.23 | 2.06 | 70 | **1.51** | 1.18 | 1.91 |
|  | Liver | 2 | 0.35 | 0.03 | 1.28 | 0 | - | - | - | 1 | 0.92 | 0 | 5.25 |
|  | Lung | 23 | 1.44 | 0.91 | 2.17 | 11 | 1.75 | 0.87 | 3.13 | 15 | **3.13** | 1.75 | 5.17 |
|  | Breast | 50 | 0.82 | 0.61 | 1.08 | 118 | **1.23** | 1.02 | 1.47 | 214 | **1.29** | 1.12 | 1.48 |
|  | Cervix | 14 | **4.47** | 2.43 | 7.52 | 11 | **3.18** | 1.58 | 5.71 | 18 | **2.43** | 1.44 | 3.85 |
|  | Endometrium | 16 | 0.97 | 0.55 | 1.58 | 36 | **1.46** | 1.03 | 2.03 | 66 | **1.54** | 1.19 | 1.96 |
|  | Ovary | 5 | 0.58 | 0.18 | 1.36 | 8 | 0.92 | 0.39 | 1.82 | 20 | **1.69** | 1.03 | 2.62 |
|  | Female genital | 6 | 2.18 | 0.79 | 4.79 | 6 | 2.29 | 0.82 | 5.01 | 4 | 1.32 | 0.34 | 3.41 |
|  | Kidney | 92 | **17.9** | 14.5 | 22.0 | 45 | **8.14** | 5.93 | 10.9 | 16 | **2.16** | 1.23 | 3.52 |
|  | RCC | 13 | **3.58** | 1.89 | 6.12 | 11 | **2.57** | 1.27 | 4.61 | 6 | 0.98 | 0.35 | 2.16 |
|  | Renal pelvis | 76 | **120.4** | 94.8 | 150.7 | 32 | **58.4** | 39.9 | 82.5 | 9 | **11.9** | 5.42 | 22.8 |
|  | Ureter | 35 | **140.1** | 97.5 | 195.0 | 9 | **38.9** | 17.6 | 74.1 | 5 | **22.5** | 7.09 | 52.8 |
|  | Melanoma | 4 | 0.45 | 0.12 | 1.17 | 16 | 1.22 | 0.69 | 1.98 | 24 | 1.10 | 0.70 | 1.64 |
|  | Skin | 16 | 0.96 | 0.55 | 1.56 | 26 | 1.28 | 0.83 | 1.87 | 19 | 1.04 | 0.62 | 1.63 |
|  | Nervous system | 4 | 0.77 | 0.20 | 1.99 | 5 | 0.83 | 0.26 | 1.95 | 19 | 1.46 | 0.88 | 2.29 |
|  | Thyroid | 0 | - | - | - | 1 | 0.50 | 0 | 2.84 | 9 | 2.04 | 0.93 | 3.90 |
|  | Endocrine | 5 | 1.03 | 0.32 | 2.41 | 9 | 1.04 | 0.47 | 1.98 | 24 | 1.39 | 0.89 | 2.07 |
|  | Connective tissue | 0 | - | - | - | 1 | 0.76 | 0 | 4.34 | 5 | 2.78 | 0.88 | 6.54 |
|  | NHL | 4 | 0.48 | 0.12 | 1.23 | 7 | 0.78 | 0.31 | 1.61 | 15 | 1.45 | 0.81 | 2.39 |
|  | Hodgkin lymphoma | 1 | 2.47 | 0 | 14.1 | 0 | - | - | - | 1 | 1.24 | 0 | 7.11 |
|  | Myeloma | 1 | 0.25 | 0 | 1.45 | 5 | 1.36 | 0.43 | 3.19 | 3 | 1.65 | 0.31 | 4.89 |
|  | Leukemia | 10 | 1.41 | 0.67 | 2.60 | 9 | 1.20 | 0.55 | 2.30 | 8 | 1.09 | 0.46 | 2.15 |
|  | ALL | 370 | **1.52** | 1.37 | 1.68 | 410 | **1.49** | 1.35 | 1.64 | 579 | **1.43** | 1.32 | 1.55 |
| **Ureter** | Lung | 2 | 3.38 | 0.32 | 12.4 | 0 | - | - | - | 0 | - | - | - |
|  | Breast | 5 | 2.41 | 0.75 | 5.66 | 1 | 0.30 | 0 | 1.74 | 11 | 1.84 | 0.91 | 3.30 |
|  | Cervix | 0 | - | - | - | 1 | 8.73 | 0 | 50.0 | 2 | 8.21 | 0.77 | 30.2 |
|  | Endometrium | 2 | 3.36 | 0.32 | 12.3 | 3 | 3.36 | 0.63 | 9.95 | 10 | **6.26** | 2.98 | 11.6 |
|  | Female genital | 0 | - | - | - | 1 | 11.3 | 0 | 65.0 | 1 | 9.38 | 0 | 53.8 |
|  | Kidney | 10 | **53.1** | 25.3 | 98.0 | 4 | **19.6** | 5.10 | 50.7 | 1 | 3.71 | 0 | 21.2 |
|  | RCC |  |  |  |  | 1 | 6.38 | 0.00 | 36.6 |  |  |  |  |
|  | Renal pelvis | 9 | **428** | 194.1 | 816.6 | 3 | **150.1** | 28.3 | 444.2 | 1 | 37.2 | 0.01 | 213.1 |
|  | Bladder | 46 | **181.0** | 132.4 | 241.5 | 16 | **53.8** | 30.6 | 87.5 | 21 | **53.4** | 33.0 | 81.8 |
|  | ALL | 71 | **8.12** | 6.34 | 10.25 | 31 | **3.14** | 2.13 | 4.46 | 57 | **3.82** | 2.89 | 4.95 |
| **Renal pelvis** | CRC | 8 | **2.90** | 1.24 | 5.74 | 8 | **3.11** | 1.33 | 6.15 | 4 | 1.13 | 0.29 | 2.93 |
|  | Lung | 1 | 0.69 | 0 | 3.95 | 1 | 0.78 | 0 | 4.48 | 3 | 1.81 | 0.34 | 5.36 |
|  | Breast | 4 | 0.88 | 0.23 | 2.28 | 3 | 0.68 | 0.13 | 2 | 6 | 1.06 | 0.38 | 2.33 |
|  | Cervix | 0 | - | - | - | 0 | - | - | - | 0 | - | - | - |
|  | Endometrium | 1 | 0.81 | 0 | 4.65 | 0 | - | - | - | 3 | 1.98 | 0.37 | 5.86 |
|  | Female genital | 1 | 4.87 | 0 | 27.92 | 1 | 5.35 | 0 | 30.64 | 0 | - | - | - |
|  | RCC | 1 | 3.27 | 0 | 18.74 | 0 | - | - | - | 0 | - | - | - |
|  | Bladder | 39 | **61.1** | 43.4 | 83.6 | 18 | **24.8** | 14.7 | 39.3 | 25 | **26.8** | 17.3 | 39.6 |
|  | Ureter | 6 | **308.4** | 111.0 | 675.8 | 0 | - | - | - | 0 | - | - | - |
|  | All | 70 | **3.11** | 2.43 | 3.94 | 48 | **1.94** | 1.43 | 2.57 | 76 | **2.15** | 1.69 | 2.69 |

SPC, second primary cancer, N= patient number, SIR standardized incidence ratio, 95%CI 95% confidence interval, RCC renal cell carcinoma, UAT, upper aerodigestive tract, CRC, colorectal cancer, NHL, non-Hodgkin lymphoma

Bolding shows that the 95%CI does not overlap with 1.00.
